# Molecular Weight Dependent Glucose Lowering Effect of Low Molecular Weight Chitosan Oligosaccharide (GO2KA1) on Postprandial Blood Glucose Level in SD Rats Model

**DOI:** 10.3390/ijms140714214

**Published:** 2013-07-09

**Authors:** Sung-Hoon Jo, Kyoung-Soo Ha, Kyoung-Sik Moon, Jong-Gwan Kim, Chen-Gum Oh, Young-Cheul Kim, Emmanouil Apostolidis, Young-In Kwon

**Affiliations:** 1 Department of Food and Nutrition, Hannam University, Daejeon 305-811, Korea; E-Mails: sunghoon04@hanmail.net (S.-H.J.); kengkoo@nate.com (K.-S.H.); 2 Division of Non-Clinical Studies, Korea Institute of Toxicology, Daejeon 305-343, Korea; E-Mail: ksmoon@kitox.re.kr; 3 Kunpoong Bio Co., Ltd., Gumnung-ri, 407-11, Hallim-eup, Jeju Special Self Governing Province, Jeju 695-923, Korea; E-Mails: hallabio@hanmail.net (J.-G.K.); cgoh@kunpoong.co.kr (C.-G.O.); 4 Department of Nutrition, University of Massachusetts, Amherst, MA 01003, USA; E-Mail: yckim@nutrition.umass.edu; 5 Department of Chemistry and Food Science, Framingham State University, Framingham, MA 01701, USA; E-Mail: eapostolidis@framingham.edu

**Keywords:** type 2 diabetes, pre-diabetes, blood glucose, α-glucosidase inhibition, low molecular chitosan oligosacharide, GO2KA1

## Abstract

This research investigated the effect of enzymatically digested low molecular weight (MW) chitosan oligosaccharide on type 2 diabetes prevention. Three different chitosan oligosaccharide samples with varying MW were evaluated *in vitro* for inhibition of rat small intestinal α-glucosidase and porcine pancreatic α-amylase (GO2KA1; <1000 Da, GO2KA2; 1000–10,000 Da, GO2KA3; MW > 10,000 Da). The *in vitro* results showed that all tested samples had similar rat α-glucosidase inhibitory and porcine α-amylase inhibitory activity. Based on these observations, we decided to further investigate the effect of all three samples at a dose of 0.1 g/kg, on reducing postprandial blood glucose levels in Sprague-Dawley (SD) rat model after sucrose loading test. In the animal trial, all tested samples had postprandial blood glucose reduction effect, when compared to control, however GO2KA1 supplementation had the strongest effect. The glucose peak (C*_max_*) for GO2KA1 and control was 152 mg/dL and 193 mg/dL, respectively. The area under the blood glucose-time curve (AUC) for GO2KA1 and control was 262 h mg/dL and 305 h mg/dL, respectively. Furthermore, the time of peak plasma concentration of blood glucose (T*_max_*) for GO2KA1 was significantly delayed (0.9 h) compared to control (0.5 h). These results suggest that GO2KA1 could have a beneficial effect for blood glucose management relevant to diabetes prevention in normal and pre-diabetic individuals. The suggested mechanism of action is via inhibition of the carbohydrate hydrolysis enzyme α-glucosidase and since GO2KA1 (MW < 1000 Da) had higher *in vivo* effect, we hypothesize that it is more readily absorbed and might exert further biological effect once it is absorbed in the blood stream, relevant to blood glucose management.

## 1. Introduction

Diabetes is a group of diseases marked by high levels of blood glucose resulting from defects in insulin production, insulin action, or both [1]. Type 2 diabetes accounts for about 90% to 95% of all diagnosed cases of diabetes in adults [1]. Pre-diabetes is a condition in which individuals have blood glucose levels higher than normal but not high enough to be classified as diabetes [2]. Pre-diabetic people have an increased risk of developing type 2 diabetes, heart disease, and stroke [3–5]. At least 220 million people worldwide have diabetes and this figure is likely to double by 2030 [6]. In the United States, in 2007, 23.7 million people (10% of American adults) had diabetes and by 2050 this figure is expected to jump to 33%, or one-third of all American adults [1]. It is important to recognize that optimal diet-induced prevention of type 2 diabetes onset is an effective strategy to reduce the expected increases in morbidity and cost associated with treatment of diabetes.

The major source of blood glucose is dietary carbohydrates that are initially hydrolyzed by pancreatic α-amylase, followed by α-glucosidase before being absorbed in the small intestine [7]. Reduction of the elevated postprandial blood glucose level is an established strategy for management of type 2 diabetes [8]. Inhibition of carbohydrate hydrolyzing enzymes, using α-glucosidase inhibitors such as Acarbose, is one way to achieve this strategy [8]. Recent studies showed that phenolic phytochemicals from botanical sources are natural inhibitors of α-amylase and α-glucosidase [9–12] and thus can be used to manage postprandial hyperglycemia with minimal side effects [9–11].

Chitosan is commercially obtained by the deacetylation of chitin, the most abundant natural biopolymer on earth after cellulose. Despite chitosan’s well-recognized beneficial effects for hypertension [13], cholesterol [14] and weight-loss [15] management, chelation of some metal ions from dietary sources becomes unfavorable for human nutrition [16]. However, these side-effects do not exist with low molecular weight chitosan oligosaccharide, resulting from the enzymatic digestion of chitosan, mainly because low molecular weight chitosan oligosaccharide can be readily absorbed into the bloodstream [16].

Low molecular weight chitosan oligosaccharide has been shown to have many health beneficial biological activities including antifungal [16], antibacterial [17–19], antitumor [20,21], immuno-enhancing [22], anti-hypertension via ACE-I inhibition [13] and protective effects against infection [23]. Additionally, Kondo and others [24] showed that low molecular weight chitosan oligosaccharide can prevent the progression of diabetes in streptozotocin-induced diabetic mice. Recently, Kim and others [25] clinically demonstrated the blood glucose lowering effect of low molecular weight chitosan oligosaccharide in healthy human subjects.

Both published studies [24,25] that investigated the effect of low molecular weight chitosan oligosaccharide for type 2 diabetes reported the observed effect without investigation the mechanism of action, which is very important for the design of more vigorous clinical studies. In this study, *in vitro* screening is used to determine the effect of different low molecular weight chitosan oligosaccharides (in terms of MW) on the inhibition of carbohydrate hydrolysis enzymes. Then all samples were further assayed *in vivo* in SD rats model for postprandial blood glucose level reduction after sucrose loading test, to further confirm the observed *in vitro* findings.

## 2. Results and Discussion

### 2.1. Rat α-Glucosidase and Porcine α-Amylase Assay

**Figure 1:**
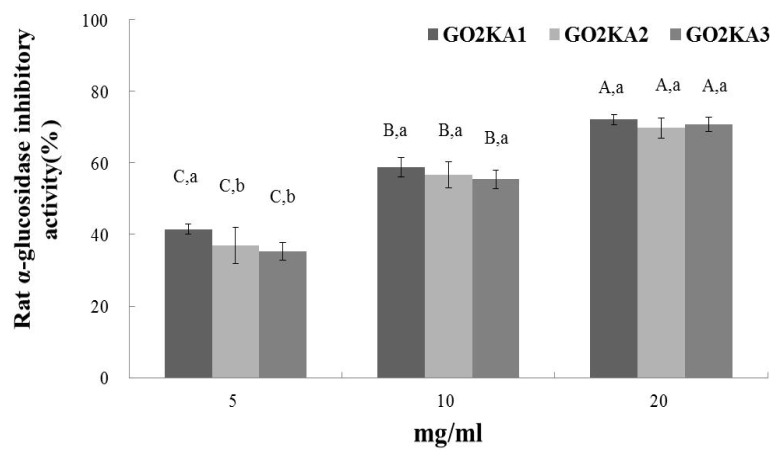
Dose dependent changes in rat intestinal α-glucosidase inhibitory activity (% inhibition) of chitosan oligosaccharides classified by molecular weight (GO2KA1; MW < 1000 Da, GO2KA2; MW 1000–10,000 Da, GO2KA3; MW > 10,000 Da). The results represent the mean ± S.D. of values obtained from three measurements. Different corresponding letters indicate significant differences at *p* < 0.05 by Duncan’s test. ^A−C^ First letter is among different samples and ^a−c^ second one is among different concentrations within same samples.

**Figure 2:**
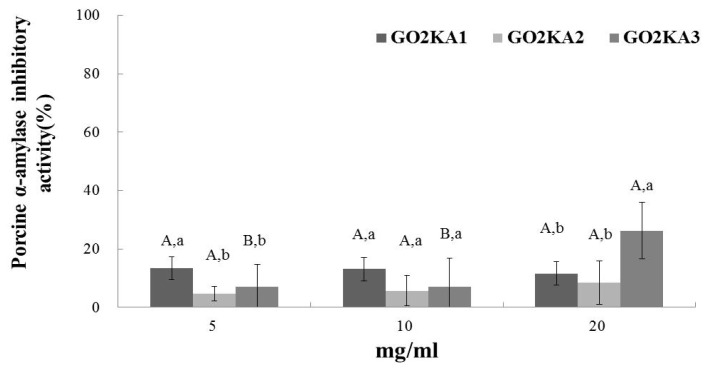
Dose dependent changes in porcine pancreas α-amylase inhibitory activity (% inhibition) of chitosan oligosaccharides classified by molecular weight (GO2KA1; MW < 1000 Da, GO2KA2; MW 1000–10,000 Da, GO2KA3; MW > 10,000 Da). The results represent the mean ± S.D. of values obtained from three measurements. Different corresponding letters indicate significant differences at *p* < 0.05 by Duncan’s test. ^A−C^ First letter is among different samples and ^a−c^ second one is among different concentrations within same samples.

All tested samples had dose-dependent and similar rat α-glucosidase inhibitory activity (Figure 1). These results indicate that the molecular weight of enzymatically digested chitosan oligosaccharide does not influence the inhibition of α-glucosidase (Figure 1). In the case of α-amylase inhibition, we observed that all the samples had significantly lower inhibitory activity, when compared to α-glucosidase inhibition (Figure 2). Similarly to α-glucosidase, it appears that the molecular weight differences do not influence the inhibitory effect of enzymatically digested chitosan oligosaccharide on α-amylase (Figure 2).

This is the first report of α-glucosidase inhibitory effect of low molecular weight chitosan oligosaccharide. Our results present a strong α-glucosidase inhibitory effect of all samples, regardless of MW, and a significantly lower α-amylase inhibitory activity. Previous reports have indicated that plant derived phenolic phytochemicals have lower α-amylase inhibitory activity and a stronger inhibition activity against α-glucosidase [10,11]. The main side effects of type 2 diabetes control drugs, such as Acarbose, are abdominal distention, flatulence, meteorism and possibly diarrhea [26]. It has been suggested that such adverse effects might be caused by the excessive inhibition of pancreatic α-amylase resulting in the abnormal bacterial fermentation of undigested carbohydrates in the colon [26,27]. Our observation of lower *in vitro* α-amylase inhibitory activity suggests that the extent of the side effects (if any) will be less than Acarbose.

### 2.2. Sucrose Loading Test in SD Rat Model

To further confirm the actual *in vivo* relevance of our *in vitro* findings that enzymatically digested chitosan oligosaccharide has α-glucosidase inhibitory effect regardless of MW, we performed a sucrose loading test in SD Rat, which is more relevant towards type 2 diabetes incidence prevention with normal or pre-diabetic individuals, rather than type 2 diabetes treatment.

**Figure 3:**
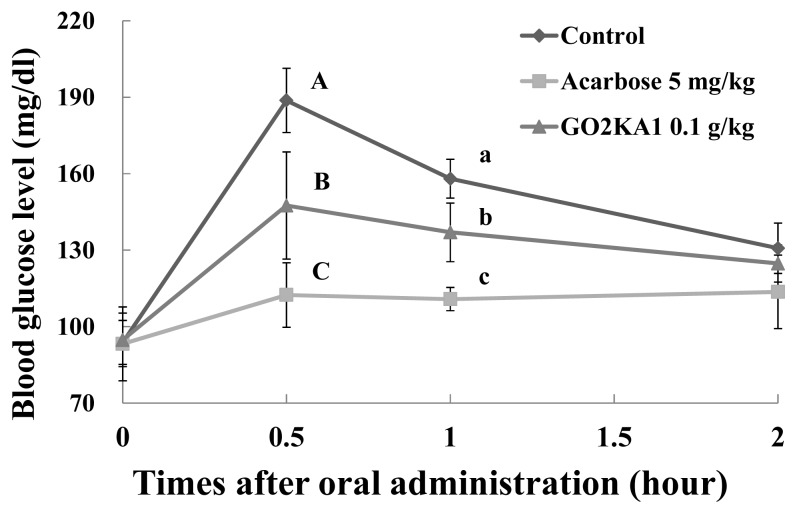
Effect of GO2KA1 on sucrose loading test. After fasting for 24 h, six-week-old, male SD rats were orally administered with sucrose solution (2.0 g/kg) with or without samples (GO2KA1; MW < 1000 Da, positive control; Acarbose). Each point represents mean ± S.D. (*n* = 5). ^A−C^ Different corresponding letters indicate significant differences (*p* < 0.05) at 0.5 h by Duncan’s test. ^a−c^ Different corresponding letters indicate significant differences (*p* < 0.05) at 1.0 h by Duncan’s test.

**Figure 4:**
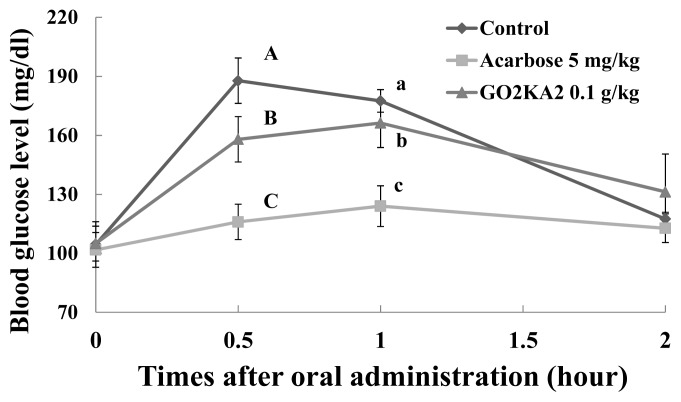
Effect of GO2KA2 on sucrose loading test. After fasting for 24 h, six-week-old, male SD rats were orally administered with sucrose solution (2.0 g/kg) with or without samples (GO2KA2; MW 1000–10,000 Da, positive control; Acarbose). Each point represents mean ± S.D. (*n* = 5). ^A−C^ Different corresponding letters indicate significant differences (*p* < 0.05) at 0.5 h by Duncan’s test. ^a−c^ Different corresponding letters indicate significant differences (*p* < 0.05) at 1.0 h by Duncan’s test.

**Figure 5:**
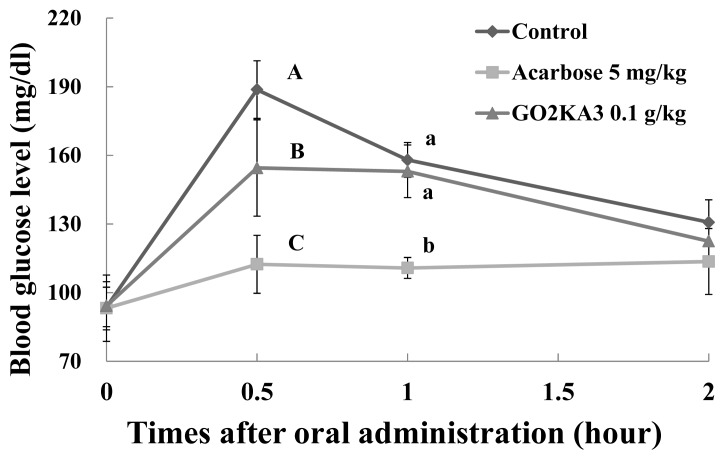
Effect of GO2KA3 on sucrose loading test. After fasting for 24 h, six-week-old, male SD rats were orally administered with sucrose solution (2.0 g/kg) with or without samples (GO2KA3; MW > 10,000 Da, positive control; Acarbose). Each point represents mean ± S.D. (*n* = 5). ^A−C^ Different corresponding letters indicate significant differences (*p* < 0.05) at 0.5 h by Duncan’s test. ^a−c^ Different corresponding letters indicate significant differences (*p* < 0.05) at 1.0 h by Duncan’s test.

Our results show that all tested samples (0.1 g/kg) result in lower blood glucose peaks when compared to control, however higher when compared to the known type 2 diabetes drug and α-glucosidase inhibitor, Acarbose (0.005 g/kg) (Figures 3–5). When we calculated the more precise pharmacodynamics of the three tested samples (Table 1), it was clear that all treatments had better effect in terms of blood glucose peak (C*_max_*), area under the curve, which represents the total amount of glucose in blood (AUC*_max_*) and the time of the observed blood glucose peak (T*_max_*), when compared to control (Table 1). Reduced blood glucose peak results in lower glucose stress that is observed after meals. Reduced AUC*_max_* indicates that either less glucose is absorbed in the blood or that glucose is more efficiently utilized when in the blood (via glucose uptake and further utilization in muscle and fat cells), or both. Finally, the retardation of T*_max_*, indicated slower absorption of glucose in the blood, which could be due to the observed *in vitro* α-glucosidase inhibitory effects.

More close evaluation of our findings suggests that GO2KA1 and GO2KA3 result in greater reduction of AUC*_max_* when compared to GO2KA2. Overall, GO2KA1 administration resulted in lower C*_max_* and AUC*_max_* values when compared to GO2KA3, but this difference was not statistically different. However, GO2KA1 administration reduced the blood glucose levels after 1 h by 14.3% (compared to control) while GO2KA3 administration resulted to a reduction around 4% (compared to control (Figures 3 and 5).

Our findings suggest that all samples have potential for lowering postprandial blood glucose levels after meal, which is relevant to type 2 diabetes incidences. Additionally, our observations, that the effect is milder when compared to Acarbose, a known drug for type 2 diabetes treatment, indicates that low molecular weight chitosan oligosaccharide can be ideal for type 2 diabetes prevention in normal and pre-diabetic individuals that do not need to use drug innovations that have severe side effects.

All enzymatically digested chitosan oligosaccharide tested samples had similar α-glucosidase inhibitory activities, which resulted in slower glucose absorption (as indicated with the similar T*_max_* values among tested samples), but GO2KA1 and GO2KA3 had better effect on AUC*_max_* (Table 1). Additionally, GO2KA1 had better effect for type 2 diabetes prevention in SD rats, in terms of blood glucose reductions of after 1 h (Figures 3 and 5), resulting to a 14%, compared to the 4% reduction resulting from GO2KA3 administration. Previous reports show that low MW chitosan oligosaccharide is more readily absorbed in the bloodstream [28,29]. We believe that low molecular weight GO2KA1 (<1000 Da) is more readily absorbed into the bloodstream when compared to the significantly larger GO2KA2 and GO2KA3 (MW 1000–10,000 Da and > 10,000 Da, respectively) and might have other possible effects towards aiding glucose absorption into muscle and fat cells. Thus, we can speculate that GO2KA1 might have a “dual” beneficial effect towards blood glucose management acting both in the gastrointestinal level, by inhibiting carbohydrate hydrolysis enzymes, as well as in the cellular level, after being absorbed into the bloodstream, by aiding towards the glucose absorption into muscle and fat cells.

## 3. Experimental Section

### 3.1. Materials

Chitosan oligosaccharides classified by molecular weight (GO2KA1; MW < 1000 Da, GO2KA2; MW 1000–10,000 Da, GO2KA3; MW > 10,000 Da) were purchased from Kunpoong Bio Co. Ltd. (Seoul, Korea). Porcine pancreatic α-amylase (EC 3.2.1.1) and rat intestinal acetone powders of α-glucosidase (EC 3.2.1.20) were also purchased from Sigma-Aldrich Co. (St. Louis, MO, USA). Unless noted, all chemicals were purchased from Sigma-Aldrich Co. (St. Louis, MO, USA).

### 3.2. α-Glucosidase Inhibition Assay

To evaluate the potency of chitosan oligosaccharide s classified by molecular weight GO2KA1, GO2KA2, GO2KA3, the dose dependent inhibitory effect of the three selected samples on rat intestinal α-glucosidase was evaluated using different concentrations (between 5 and 20 mg/mL). Rat intestinal α-glucosidase assay referred to the method of Kwon *et al.* [10] with slight modification. A total of 0.1 g of rat-intestinal acetone powder was suspended in 3 mL of 0.9% saline, and the suspension was sonicated 12 times for 30 s at 4 °C. After centrifugation (10,000*g*, 30 min, 4 °C), the resulting supernatant was used for the assay. Sample solution (50 μL) and 0.1 M phosphate buffer (pH 6.9, 100 μL) containing glucosidase solution (1.0 U/mL) was incubated at 25 °C for 10 min. After pre-incubation, 5 mM *p*-nitrophenyl-α-d-glucopyranoside solution (50 μL) in 0.1 M phosphate buffer (pH 6.9) was added to each well at timed intervals. The reaction mixtures were incubated at 25 °C for 5 min. Before and after incubation, absorbance was read at 405 nm and compared to a control, which had 50 μL of buffer solution in place of the extract by micro-plate reader (SUNRISE; Tecan Trading AG, Saltzburg, Austria). The α-glucosidase inhibitory activity was expressed as inhibition % and was calculated as follows (1):

(1) $$\text{Inhibition }(\%)=\left(\left[\frac{\Delta A_{405}^{Control}-\Delta A_{405}^{Extract}}{\left[\Delta A_{405}^{Control}\right]}\right]\right)\times 100$$

### 3.3. a-Amylase Inhibition Assay

To evaluate the potency of chitosan oligosaccharide s classified by molecular weight (GO2KA1, GO2KA2, GO2KA3) the dose dependent inhibitory effect of the three selected samples on rat intestinal α-amylase was evaluated using different concentrations (between 5 and 20 mg/mL). Porcine pancreatic α-amylase inhibition referred to the method of Kwon *et al.* [10]. Sample solution (200 μL) and 0.02 M sodium phosphate buffer (pH 6.9 with 0.006 M sodium chloride, 500 μL) containing α-amylase solution (0.5 mg/mL, 5.0 mU/mL) were incubated at 25 °C for 10 min. After pre-incubation, 500 μL of a 1% starch solution in 0.02 M sodium phosphate buffer was added. The reaction mixture was then incubated at 25 °C for 10 min. The reaction was stopped with 1.0 mL of dinitrosalicylic acid (DNS) and incubation in a boiling water bath for 5 min, followed by cooling down to room temperature. The reaction mixture was then diluted after adding distilled water, and absorbance was measured at 540 nm (2) with ELISA micro-plate reader (SUNRISE; Tecan Trading AG, Saltzburg, Austria).

(2) $$\text{Inhibition }(\%)=\left(\left[\frac{\Delta A_{540}^{Control}-\Delta A_{540}^{Extract}}{\left[\Delta A_{540}^{Control}\right]}\right]\right)\times 100$$

### 3.4. Sugar Loading Test

Effect on hyperglycemia induced by carbohydrate loads in Sprague-Dawley (SD) rats was evaluated by the studying the inhibitory action of GO2KA1, GO2KA2, GO2KA3 and Acarbose on postprandial hyperglycemia. Five week-old male SD rats were purchased from Joongang Experimental Animal Co. (Seoul, Korea) and fed a solid diet (Samyang Diet Co., Seoul, Korea) for one week. The rats were housed in a ventilated room at 25 ± 2 °C with 50% ± 7% relative humidity, and under an alternating 12 h light/dark cycle. After six groups of five male SD rats (180–200 g) were fasted for 24 h, 2.0 g/kg of sucrose were orally administrated concurrently with 0–500 mg/kg inhibitors (GO2KA1, GO2KA2, GO2KA3 and Acarbose). The blood samples were then taken from the tail after administration and blood glucose levels were measured at 0, 0.5, 1, and 2 h. The glucose level in blood was determined by glucose oxidase method and compared with that of the control group, which had not taken the inhibitors. The parameters for blood glucose levels were calculated using WinNonLin program (Version 5.2.1, Pharsight Corporation, Cary, NC, USA). Maximum observed peak blood glucose level (C*_max_*) and the time at which it is observed (T*_max_*) were determined based on the observed data. Area under the blood glucose-time curve up to the last sampled time-point (AUC*_last_*) was estimated by the trapezoidal rule.

### 3.5. Statistical Analysis

All data are presented as mean ± S.D. Statistical analyses were carried out using the statistical package SPSS (Statistical Package for Social Science, SPSS Inc., Chicago, IL, USA) program and significance of each group was verified with the analysis of One-way ANOVA followed by the Duncan’s test of *p* < 0.05.

## 4. Conclusions

This is the first report of low molecular weight chitosan oligosaccharide mediated α-glucosidase inhibition, relevant to type 2 diabetes prevention. Our significant *in vitro* and *in vivo* findings indicate that enzymatically digested chitosan oligosaccharide has α-glucosidase inhibitory activity, which is not molecular weight dependent. Our *in vivo* observations suggest that all tested samples had significant and similar effect on blood glucose management in the tested model. However, GO2KA1, with MW < 1000 Da seemed to have a better effect towards postprandial blood glucose management after 1 h of meal. This observation should not be overlooked and further evaluated. We hypothesize, that GO2KA1 with MW below 1000 Da might have “dual” effect towards glucose management by inhibiting carbohydrate hydrolysis enzymes, as well as by aiding towards the glucose absorption into muscle and fat cells in the cellular level, since it is easier absorbed into the bloodstream (when compared to the significantly larger GO2KA2 and GO2KA3). Based on our findings more research will be performed towards elucidating the exact mechanism of action of GO2KA1 for type 2 diabetes prevention, with focus on the potential insulin sensitizing effect on muscle and fat cells.

## Figures and Tables

**Table 1:** Pharmacodynamic (PD) parameters of SD control rats or after administration of GO2KA1, GO2KA2, GO2KA3 and Acarbose after sucrose ingestion.

Groups		PD parameters		

		AUC*_last_* (h·mg/dL)	C*_max_* (mg/dL)	T*_max_* (h)
Sucrose	Control	305.1 ± 7.3 A	193.0 ± 5.1 a	0.5 ± 0.0
	Acarbose (5.0 mg/kg)	214.7 ± 15.2 B	118.8 ± 10.9 b	1.2 ± 0.8
	GO2KA1 (0.1 g/kg)	262.4 ± 11.9 C	152.0 ± 6.3 c	0.9 ± 0.7

		**PD parameters**		
		
		**AUC***_last_***(h**·**mg/dL)**	**C***_max_***(mg/dL)**	**T***_max_***(h)**

Sucrose	Control	306.8 ± 7.3 A	192.0 ± 8.8 a	0.6 ± 0.2
	Acarbose (5.0 mg/kg)	226.1 ± 5.8 B	125.2 ± 7.0 b	0.9 ± 0.2
	GO2KA2 (0.1 g/kg)	289.1 ± 10.1 A	167.0 ± 12.8 c	0.9 ± 0.2

		**PD parameters**		
		
		**AUC***_last_***(h**·**mg/dL)**	**C***_max_***(mg/dL)**	**T***_max_***(h)**

Sucrose	Control	305.1 ± 7.3 A	193.0 ± 5.1 a	0.5 ± 0.0
	Acarbose (5.0 mg/kg)	222.5 ± 17.8 B	116.4 ± 10.8 b	1.2 ± 0.8
	GO2KA3 (0.1 g/kg)	268.3 ± 11.2 C	154.0 ± 9.2 c	0.7 ± 0.3

A−C & a−c Different corresponding letters indicate significant differences at *p* < 0.05 by Duncan’s test.
